# A descriptive study of variables associated with obtaining nipple aspirate fluid in a cohort of non-lactating women

**DOI:** 10.1186/1472-6874-6-15

**Published:** 2006-10-17

**Authors:** Kimberly A Baltzell, Margaret Wrensch, Jennette D Sison

**Affiliations:** 1 University of California, San Francisco Department of Physiological Nursing San Francisco, CA, USA; 2 University of California, San Francisco Department of Neurological Surgery San Francisco, CA, USA

## Abstract

**Background:**

The search for biologic endpoints and biomarkers in the study of breast cancer risk assessment and risk reduction strategies has led to an interest in obtaining cytologic information and other biomarkers from nipple aspirate fluid (NAF).

**Methods:**

This descriptive study examined factors associated with an increased ability to obtain NAF in a cohort of 3043 women between the ages of 15 and 89 years of age. The majority of women were between the ages of 30–49 (N = 1529/50.2%). Variables examined in relation to obtaining fluid include: age, marital status, age at menarche, menopausal status, a history of pregnancy, a history of breast-feeding, estrogen use, oral contraceptive use, endocrine disorders and tranquilizer use.

**Results:**

On average, women from whom breast fluid was obtained were younger than women from whom breast fluid was attempted but not obtained (mean = 41.9 years versus 46.5 years, p < 0.0001). In unadjusted and age-adjusted comparisons, being married, a history of pregnancy, younger age at menarche (12 years of age or younger), tranquilizer use, oral contraceptive pill (OCP) use and endocrine problems were associated with an increased ability to obtain breast fluid. Post-menopausal status and exogenous estrogen use were associated with a decreased ability to obtain breast fluid. After age-adjustment, oral contraceptive use was no longer significantly associated with an increased ability to obtain fluid and post-menopausal status was no longer associated with a decreased ability to obtain breast fluid. After multivariate adjustment, age, being married, a history of pregnancy, tranquilizer use and a history of endocrine problems remained positively associated with the ability to obtain breast fluid. In addition, menopausal women who took estrogen were less likely to yield fluid than premenopausal women.

**Conclusion:**

Four variables (being married, history of pregnancy, tranquilizer use and endocrine disorders) remained positively associated with the ability to obtain NAF in all analyses. A younger age was consistently associated with a greater ability to obtain NAF in this and other studies.

## Background

The search for early stage breast cancer, or more importantly, its precursors, is the goal of many researchers hoping to reduce mortality rates from this disease. Current methods of breast cancer early detection include mammography, ultrasound, clinical breast exam (CBE) and breast self-examination (BSE). In addition to these methods, other imaging techniques such as magnetic resonance imaging (MRI) are being explored [1]. Although these cancer detection methods have been used successfully for specific populations, biomarkers that come directly from the breast cells may add to the information obtained from the above-mentioned detection methods. One of the commonly used detection methods, mammography, appears to be less sensitive for younger women (<55 years) [2]. Despite high suspicion of malignancy in mammography, CBE or BSE, a breast cancer diagnosis cannot be made definitively without tissue or cytologic confirmation. More importantly, concentrating on precursors of breast cancer would allow for intervention prior to the development of a malignancy. Intermediate biologic endpoints are also necessary for the evaluation of chemopreventive therapies [3]. Recent studies have indicated that cells from nipple aspirate fluid (NAF) may yield useful information for enhancing breast cancer risk assessment [4]. However, sensitivity has been found to be low in recent studies using other methods of breast fluid analysis for detection of breast cancer, including peri-areolar fine needle aspiration and ductal lavage [5,6]. Determining which populations will derive the most benefit from the analysis of breast fluid requires additional study of the characteristics of the women most likely to yield fluid resulting in an accurate cytologic diagnosis. Analyzing cells in NAF is a step toward identifying tissue and/or serum based markers of breast cancer risk as 70% of women who develop breast cancer have no identifiable risks factors [7].

In order to utilize NAF as both an adjunct to current breast cancer risk assessment tools and/or for breast cancer early detection, it is important to understand factors influencing the obtainment of adequate fluid samples. Past studies have shown four factors consistently associated with an increased ability to obtain breast fluid; age between 35–50 years, earlier age at menarche, non-Asian compared to Asian ethnicity and a history of lactation [8]. Using a descriptive cross-sectional design, this study examined demographic, menstrual, reproductive and other factors in relation to the ability to obtain NAF in a group of 3043 non-lactating women from the Santa Barbara, California area.

## Methods

### Subjects

All women were volunteers seen in a private breast clinic by one physician, Dr. Otto Sartorius, who attempted NAF collection on 3413 women. The women came to the clinic for a variety of reasons and NAF was attempted as part of Dr. Sartorius's normal evaluation. The volunteers consisted of women without known breast disease, as well as private clinic patients with known or suspected breast disease [9]. For this paper, all women with breast cancer or women who were diagnosed with breast cancer within six months of their initial clinic visit were excluded from the analysis, reducing the number of eligible subjects to 3043. Dr. Sartorius was a pioneer in the field of breast cancer research, conducting multiple studies in the area of NAF. Upon his death in 1994, Dr. Susan Love was named medical director of his research foundation. The records of Dr. Sartorius are held by The Dr. Susan Love Research Foundation, which granted access to the records to this study's first author, Kimberly Baltzell. Data regarding the obtainment of NAF and medical, reproductive and other factors were abstracted from medical records by a team led by Sandra Tillisch, R.N. in Santa Barbara, California. Human subjects approval was obtained from University of California, San Francisco (UCSF) Committee on Human Research and the UCSF Protocol Review Committee (PRC).

### NAF collection

The nipple aspiration technique utilized on this cohort was developed by Dr. Otto Sartorius [9]. Dr Sartorius performed all NAF attempts on this cohort for the specified time period. The aspiration device used was double-chambered. The nipples were scrubbed with 2% acetic acid to remove encrustations, and then dried before attempting aspiration. The aspiration device was placed over the nipple and negative pressure was applied with a syringe attached to the central chamber. If the central chamber was occluded, negative pressure was applied first to the outer chamber. Once fluid was observed, the aspiration device was removed and the fluid was collected in capillary tubes. This procedure was repeated until no additional fluid was expressed. Any visible or measurable quantity of NAF was considered a positive NAF sample. Volume varied from < 1 microliter to greater than 50 microliters. Past studies have shown an increase in cytologic adequacy with an increase in NAF volume [9].

### Statistical analysis

Statistical Analysis Software (SAS) [10] version 8.02 was used for all statistical analyses. Distributions of variables from women for whom NAF was obtained were compared to women from whom NAF collection was attempted but not obtained using SAS procedures FREQ or UNIVARIATE and p-values were determined from chi-square and t-tests for discrete and continuous variables, respectively. Logistic regression analysis was used to estimate odds ratios for obtaining versus not obtaining fluid for variables individually adjusted for age and in a multivariate model that included age, marital status, age at menarche, pregnancy history, estrogen use, tranquilizer use and endocrine problems using SAS, PROC LOGISTIC.

## Results

The women seen at the clinic were between the ages of 15–89 years at the time of breast fluid extraction. Although ethnicity data were only recorded for 16% of the cohort, the geographic area is predominantly Caucasian. Of the ethnicity information known for 16% of this cohort, 89.1% of those were Caucasian (N = 443). Cohort demographics are listed in Table 1.

**Table 1:** Demographic, menstrual, reproductive, and other characteristics of women from the Sartorius Nipple Aspirate Fluid Cohort, 1970–1990.

**Characteristic Variables**	**N (#)**	**%**	**Median**	**Mean**	**SE**
Continuous					
**Age (years)**	3043		43	44.5	0.27
**Height (inches)**	2927		64.5	64.5	0.05
**Earliest Weight (lbs.)**	2921		130	136.4	0.49
**Body Mass Index**	2910		22.3	23.4	0.08
					
Discrete					
**Marital Status**					
Married	1833	60.2			
Single	530	17.4			
Divorced	307	10.1			
Widowed	178	5.9			
No Data	195	6.4			
					
**Age at Menarche (yrs)**					
< 10 yrs	25	0.8			
10 to 12 yrs	945	31.1			
13 to 14 yrs	1259	41.4			
> 14 yrs	317	10.4			
No Data	497	16.3			
					
**History of Pregnancy**					
Yes	2155	70.8			
No	887	29.2			
Missing	1	0			
					
**Breastfed Any Child**					
Yes	1209	39.7			
No, but history of pregnancy	870	28.6			
Never pregnant	887	29.2			
Missing	76	2.5			
					
**Menopausal**					
Yes	1328	43.6			
No	1600	52.6			
No Data	115	3.8			
					
**Age at Menopause (yrs)**					
20 to 29 yrs	98	3.2	7.4*		
30 to 39 yrs	297	9.8	22.4*		
40 to 49 yrs	498	16.4	37.5*		
50 to 59 yrs	401	13.2	30.2*		
> 59 yrs	6	0.2	0.5*		
Premenopausal	1600	52.6			
No Data	143	4.7			
					
**Menopause Reason**					
Natural menopause	500	16.4	37.7^†^		
Hysterectomy only	419	13.8	31.6^†^		
Hysterectomy + 1 ovary	79	2.6	5.9^†^		
Hysterectomy + both ovaries	256	8.4	19.3^†^		
Both ovaries removed only	2	0.1	0.2^†^		
Radiation	3	0.1	0.2^†^		
Chemotherapy	2	0.1	0.2^†^		
Hysterectomy, ovary unkn	4	0.1	0.3^†^		
Premenopausal	1600	52.6			
No Data	178	5.8			
					
**External Estrogen (among menopausal women only n = 1328)**					
Yes	838	63.1			
No	466	35.1			
No Data	24	1.8			
					
**Tranquilizer Use**					
Yes	365	12			
No	2510	82.5			
No Data	168	5.5			
					
**Oral Contraceptive Pills**					
Yes	1580	51.8			
No	1124	36.9			
No Data	339	11.1			
					
Endocrine Problems**	508	16.7			
Yes	2393	78.6			
No	142	4.7			
No Data					

SE, standard error of the Mean

* Percentage is among 1328 menopausal women with 28 missing age at menopause.

^† ^Percentage from 1328 menopausal women with 63 missing menopause reason.

** Among women with endocrine problems 61 had diabetes, 21 had hyperthyroidism, 382 had hypothyroidism, and other types of endocrine problems are unknown.

**Figure 1:**
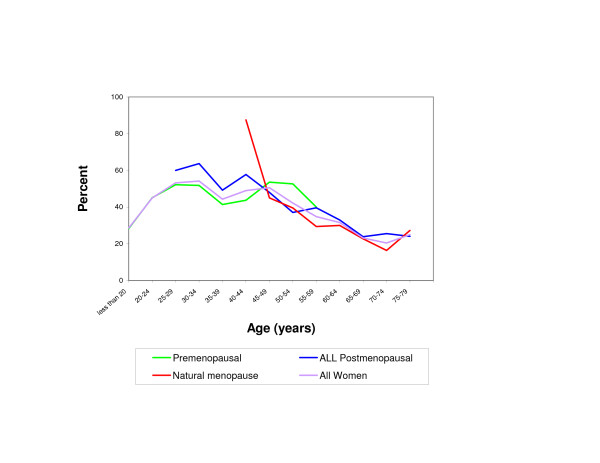
Percent of women yielding fluid by menopausal status and 5-year age groups; Sartorius Nipple Aspirate Cohort, 1970–1990. * natural menopause is defined as menopause that was not induced by chemotherapy, radiation or ovarian ablation.

Fluid was obtained from 1314 women (43%) and attempted but not obtained from 1729 women (57%). Overall, the mean age for women who yielded breast fluid was 41.9 years versus 46.5 years for women from whom aspiration was unsuccessfully attempted (p=<.0001). NAF production is lower in women under 30, and then relatively constant for the next 25 years, and then declines in older age women (Figure 1.) Figure 1 highlights the trend in breast fluid production in women by 5-year age intervals. A decrease in the ability to obtain fluid is evident in all categories (premenopausal, natural menopause and all postmenopausal) beginning at approximately age 55.

In both unadjusted and age-adjusted comparisons, fluid was more likely to be obtained from women who were married, had a history of any pregnancy, tranquilizer use, a younger age at menarche, or endocrine problems (defined as hypothyroidism, hyperthyroidism or diabetes). Fluid was less likely to be obtained from women who used exogenous estrogen (Table 3). After multivariate adjustment, women who were married, had a history of pregnancy, tranquilizer use and endocrine problems remained significantly more likely to yield breast fluid, while a younger age at menarche was no longer significantly associated. In addition, menopausal women who took estrogen were less likely to yield fluid than premenopausal women. Adding body mass index (BMI) to the multivariate analysis did not influence significant factors appreciably, and therefore, was excluded from the final analysis. A history of breast-feeding was not significant in any of the analyses.

**Table 2:** Comparison of continuous variable mean and medians for women from whom fluid was obtained versus not obtained, Sartorius Nipple Aspirate Fluid Cohort, 1970–1990.

	**NAF Fluid Obtained**				**NAF Fluid NOT Obtained**				
***Continuous Variables***	**N (#)**	**Median**	**Mean**	SE*	**N (#)**	**Median**	**Mean**	**SE**	**t-test p-value**

**Age (years)**	1314	41.0	41.9	0.36	1729	45.0	46.5	0.38	***<0.0001***
**Height (inches)**	1249	65.0	64.6	0.07	1678	64.5	64.5	0.07	0.87
**Earliest Weight (lbs.)**	1242	130.0	134.9	0.75	1679	132.0	137.4	0.64	***0.002***
**Body Mass Index**	1239	22.1	23.1	0.12	1671	22.4	23.6	0.11	***0.001***

* SE, standard error of the Mean

**Table 3:** Unadjusted, adjusted, and multivariate analyses of factors influencing the ability to obtain nipple aspirate fluid (NAF), Sartorius Nipple Aspirate Fluid Cohort, 1970–1990.

	**No NAF Obtained**	**NAF Obtained**	**Chi-square**	**Age-adjusted Odds**				Multivariate* adjusted Odds			
**VARIABLE**	**N (%)**	**N (%)**	**p-value**	**Ratio**	95% CI*		**p-value**	**Ratio**	**95% CI**		**p-value**

**Marital Status**											
No	617 (60.8)	398 (39.2)									
Yes	988 (53.9)	845 (46.1)	**0.0004**	**1.39**	**1.19**	**1.63**	**<0.0001**	**1.30**	**1.06**	**1.59**	**0.01**
											
**Age at Menarche**											
13 and older	981 (62.3)	595 (37.8)	**0.004**	**1.19**	**1.01**	**1.41**	**0.04**	1.18	0.98	1.43	0.08
12 or younger	548 (56.5)	422 (43.5)									
											
**Pregnancy History**											
Never pregnant	546 (61.6)	341 (38.4)									
Ever pregnant	1183 (54.9)	972 (45.1)	**0.0007**	**1.66**	**1.40**	**1.97**	**<0.0001**	**1.55**	**1.24**	**1.94**	**0.0001**
											
**Breastfed Children**											
Never Breastfed	483 (55.5)	387 (44.5)									
Ever Breastfed	663 (54.8)	546 (45.2)	0.76	0.96	0.80	1.15	0.65				
											
**Menopause**											
No	845 (52.8)	755 (47.2)									
Yes	838 (63.1)	490 (36.9)	**<0.0001**	1.04	0.84	1.29	0.73				
											
**Estrogen Use**											
No	1116 (56.1)	755 (47.2)									
Yes	580 (63.2)	489 (36.9)	**0.02**	**1.23**	**1.02**	**1.48**	**0.03**				
											
**Menopausal status and Estrogen use**											
Premenopausal women	822 (53.3)	721 (46.7	**<0.0001**	referent				referent			
Menopausal and NO estrogen	293 (62.9)	173 (37.1)		1.00	0.77	1.29	0.99	0.76	0.55	1.04	0.09
Menopausal and estrogen	538 (64.2)	300 (35.8)		1.01	0.79	1.28	0.94	**0.71**	**0.52**	**0.95**	**0.02**
											
**Oral Contraceptive Pills**											
No	715 (63.6)	409 (36.4)									
Yes	873 (55.3)	707 (44.8)	**<0.0001**	0.98	0.82	1.17	0.82				
											
**Tranquilizer Use**											
No	1501 (59.8)	1009 (40.2)									
Yes	145 (39.7)	220 (60.3)	**<0.0001**	**2.50**	**2.00**	**3.15**	**<0.0001**	**2.20**	**1.63**	**2.98**	**<0.0001**
											
**Endocrine Problems**											
Never	1401 (58.6)	992 (41.5)									
Ever had	273 (53.7)	235 (46.3)	**0.05**	**1.47**	**1.21**	**1.80**	**0.0001**	**1.39**	**1.07**	**1.81**	**0.01**

* Multivariate model is adjusted for age, marital status, age at menarche, any pregnancy history, the combination of menopausal status and estrogen use, tranquilizer use, and endocrine problems (in the multivariate model N = 2104)

** CI, Confidence interval

Total number of women for whom NAF fluid was not obtained = 1729 (56.8%)

Total number of women for whom NAF fluid was obtained = 1314 (43.2%)

Total number of women, N = 3043

## Discussion

This study builds on the work of Wrensch et al. [8] and others in illuminating factors associated with an increased ability to obtain NAF. Wrensch et al.'s work compared findings from 5 previous studies with the findings from a cohort of 1428 women. Four factors were consistently associated with NAF obtainment in the comparison: 1) age 35–55 years, 2) an earlier age at menarche, 3) non-Asian compared to Asian ethnicity and 4) history of lactation. In the present study, younger age was also found to be positively associated with obtaining fluid. This is consistent with findings by Wrensch et al. [8], Petrakis et al. [11], Petrakis et al. [12] and Wnyder et al. [13]. Wrensch et al. [8] suggested that a decline in secretory activity of the breast may explain these findings, rather than the onset of menopause.

Although being married was positively associated in multivariate analysis with increased ability to obtain fluid in the present study, it was examined in only one other comparison study [14] and was not found to be significant. Marital status remained significant in all analyses in the present study, including adjustment for age and a history of pregnancy, discounting the speculation that confounding of marriage with parity and breast-feeding might explain the increased ability to obtain fluid. Given there are no ready biologic reasons to explain this finding, additional study of this variable is warranted.

A previous history of pregnancy was highly significant in the present study in all analyses and this is consistent with findings from Buehring [15] and Petrakis et al. [12]. Previous studies [8,12,15], found an increased ability to obtain breast fluid in women with a history of lactation, although this study did not.

In the present study, postmenopausal women who used estrogen were significantly less likely to yield fluid compared with premenopausal women. Menopausal estrogen use was not found to be significant in any of the previous comparison studies [12-16] with the exception of Petrakis et al[12], who found that women after age 60 who used estrogen were significantly more likely to yield fluid than women who did not use estrogen over the age of 60. This is in direct contrast to the present study findings; however, this study did not differentiate estrogen users by age, only menopausal status. Given the lack of significant findings in other similar studies, it appears that exogenous estrogen use in post-menopausal women may not be an important determinant of ability to obtain breast fluid. In fact, in the present study, the inverse was found. In addition, the present study found that there were no significant differences between women who were post-menopausal and did not take estrogen and premenopausal women after adjusting for age. It seems likely that endogenous reproductive and menstrual hormones are partly responsible for the greater ability to obtain NAF in younger versus older women or in women with intact ovaries versus those with bilateral oophorectomy, given recent studies showing reduced NAF yield in women undergoing bilateral salpingo-oophorectomy (BSO), making rates of NAF production for premenopausal women undergoing BSO similar to the rates of post-menopausal women [17]. However, at a certain age (which likely varies between women), aspects of senescence other than declining endogenous hormones also might influence breast physiology, the production of NAF, and the ability to obtain it. Greater understanding of these factors would be necessary to explain why exogenous estrogens do not appear to increase ability to obtain NAF.

Tranquilizer use was positively associated with an increased ability to yield breast fluid; however, this factor was not found to be significant in the only comparison study that considered this factor [12]. Sartorius [18] suggested that some tranquilizers appear to increase breast secretions but did not formally study the effects for statistical significance. Looking at the pharmaceutical properties of tranquilizers will be necessary to further ascertain the role of this type of medication in increasing breast fluid production. The tranquilizers used during this period were probably different from tranquilizers that are used currently; therefore, these findings may not be applicable to today's women. In addition, tranquilizers may alter the protein and cellular content of NAF, affecting the fluid biology and its diagnostic value.

The endocrine disorders studied from the Sartorius cohort produced conflicting findings, given that the disorders included are diabetes and both hyper- and hypo-thyroidism. Wynder et al. [14] studied similar endocrine disorders but did not find any significant associations with an increased breast fluid yield.

Factors which were not found to be significantly associated with breast fluid yield in the present study which are consistent with lack of significance in other studies include menopausal status and oral contraceptive use. Only one study found oral contraceptive use to be significantly related to an increased ability to obtain breast fluid [15]. The following significant findings in the present study; marital status, tranquilizer use and a history of endocrine disorders, have not been widely examined in other nipple aspirate fluid studies.

While the Sartorius cohort represents a unique group from which to examine important factors related to obtaining NAF, the data do have some limitations. Most notable is that in contrast to other studies, a structured questionnaire was not used to obtain information on potentially relevant factors. Instead, the data were obtained from medical record abstraction. It is possible that there may have been some inconsistency over time in how or whether factors were recorded in the medical records. Also, the cohort included women with suspected breast disease; therefore, the ability to generalize the findings may be limited. All available information regarding the characteristics of the cohort was disclosed in Table 1. Differences between studies in collection of risk factor data and characteristics of the women themselves may explain some of the inconsistencies between studies. In addition, Dr Sartorius was the sole collector of NAF in this study. Since 1990, the end point for the Sartorius study data collection, other NAF collection devices have been developed and different collection techniques have been tried. The technique mostly commonly used now includes applying warm compresses to the breast, gentle massage and the use of an aspiration cup (FirstCyte aspirator; Cytyc Corporation, Marlborough, MA) [19]. NAF collection rates may be influenced by the device used, the technique used, as well as the person performing the NAF collection. In a 1990 meta-analysis, differences were noted in maximum pressure used, duration of pressure and definition of secretor status [8]. All of these factors impact the percentage of successful NAF attempts reported. The quantity of the fluid present, the characteristics of the duct and nipple and the proficiency of the investigator collecting the specimen (which appears to increase after > 30 different individual NAF collections) also influence the success rates reported [4]. Regardless of technique used, collection rates are consistently higher in younger women.

## Conclusion

Taken together, this and other studies suggest that age is the most consistent factor affecting obtainment of breast fluid. Wrensch et al. [4,8] obtained NAF from 42% of women less than 50 years of age. This study found NAF was obtained from roughly half of all women under 50 years, decreasing markedly after age 50 (49% and 26% respectively). Nipple aspirate fluid analysis may hold promise for younger women who do not fully benefit from current breast cancer risk assessment tools and/or breast cancer detection methods. More studies evaluating the feasibility of examining breast cells from NAF, as well as clarifying the most likely population for its use, are necessary.

## Abbreviations

BMI – Body mass index

BSO – Bilateral salpingo-oophorectomy

BSE – Breast self-examination

CBE – Clinical breast exam

MRI – Magnetic resonance imaging

NAF – Nipple aspirate fluid

OCP – Oral contraceptive pills

PRC – Protocol Review Committee

SAS – Statistical Analysis Software

UCSF – University of California San Francisco

## Competing interests

The author(s) declare that they have no competing interests.

## Authors' contributions

KB conceived of the study and set up the design and coordination of the research team. She also reviewed and interpreted all statistical analyses and drafted the original manuscript. MW participated in the study design and helped to draft the manuscript, including reviewing data analysis and providing assistance with revisions. JS performed statistical analyses. All authors have read and approved the final manuscript.

## Pre-publication history

The pre-publication history for this paper can be accessed here:



## References

[B1] Hollingsworth AB, Stough R (2003). The emerging role of breast magnetic resonance imaging. The Journal of the Oklahoma State Medical Association.

[B2] Kolb T, Lichy J, Newhouse J (2002). Comparison of the performance of screening mammography, physical examination, and breast US and evaluation of factors that influence them: an analysis of 27,825 patient evaluations. Radiology.

[B3] Daly M, Ross E (2000). Predicting breast cancer: the search for a model. Journal of the National Cancer Institute.

[B4] Wrensch MR, Petrakis NL, Miike R, King EB, Chew K, Neuhaus J, Lee MM, Rhys M (2001). Breast cancer risk in women with abnormal cytology in nipple aspirates of breast fluid. Journal of the National Cancer Institute.

[B5] Khan SA, Wiley EL, Rodriquez N, Baird C, Ramakrishnan R, Nayar R, Bryk M, Bethke K, Staradub VL, Wolfman J, Rademaker A, Ljung BM, Morrow M (2004). Ductal lavage findings in women with known breast cancer undergoing mastectomy. Journal of the National Cancer Institute.

[B6] Fabian CJ, Kimler BF, Mayo MS (2004). Ductal lavage for early detection - what doesn't come out in the wash. Journal of the National Cancer Institute.

[B7] Hollingsworth AB, Nall S, Dill D (2002). The evolution of breast cancer risk assessment. Journal of the Oklahoma State Medical Association.

[B8] Wrensch MR, Petrakis NL, Gruenke LD, Ernster VL, Miike R, King EB, Hauck WW (1990). Factors associated with obtaining nipple aspirate fluid: analysis of 1428 women and literature review. Breast Cancer Research and Treatment.

[B9] Sartorius O, Smith H, Morris P, Benedict D, Friesen L (1977). Cytologic evaluation of breast fluid in the detection of breast disease. Journal of the National Cancer Institute.

[B10] SAS Institute (1990). SAS Procedures Guide.

[B11] Petrakis NL, Mason L, Lee R, Sugimoto B, Pawson S, Catchpool F (1975). Association of race, age, menopausal status, and cerumen type with breast fluid secretion in nonlactating women, as determined by nipple aspiration.. Journal of the National Cancer Institute.

[B12] Petrakis NL, Ernster VL, Sack S, King EB, Schweitzer R, Hunt T, King M (1981). Epidemiology of breast fluid secretion: association with breast cancer risk factors and cerumen type. Journal of the National Cancer Institute.

[B13] Wynder E, Lahti H, Laakso K, Cheng SL, DeBevoise S, Rose D (1985). Nipple aspirates of breast fluid and the epidemiology of breast disease.. Cancer.

[B14] Wynder E, Hill P, Laakso K, Littner R, Kettunen K (1981). Breast secretion in Finnish women: A metabolic epidemiologic study. Cancer.

[B15] Buehring G (1979). Screening for breast atypias using exfoliative cytology.. Cancer.

[B16] Sharma P, Klemp JR, Simonsen M, Welsko C, Zalles CM, Kimler BF, Fabian CJ (2004). Failure of high risk women to produce nipple aspirate fluid does not exclude detection of cytologic atypia in random periareolar fine needle aspiration specimens.. Breast Cancer Research and Treatment.

[B17] Higgins S, Matloff E, Rimm D, Dziura J, Haffty BG, King BL (2005). Patterns of reduced nipple aspirate fluid production and ductal lavage cellularity in women at high risk for breast cancer. Breast Cancer Research and Treatment.

[B18] Sartorius O (1973). Breast fluid cells help in early cancer detection.. Journal of the American Medical Association.

[B19] Dua S, Isacke C, Gui G (2006). The intraductal approach to breast cancer biomarker discovery. Journal of Clinical Oncology.

